# Communicable Diseases Prioritized According to Their Public Health Relevance, Sweden, 2013

**DOI:** 10.1371/journal.pone.0136353

**Published:** 2015-09-23

**Authors:** Viktor Dahl, Anders Tegnell, Anders Wallensten

**Affiliations:** 1 The Public Health Agency of Sweden, Stockholm, Sweden; 2 European Programme for Intervention Epidemiology Training (EPIET), European Centre for Disease Prevention and Control (ECDC), Stockholm, Sweden; 3 Department of Medical Sciences, Uppsala University, Uppsala, Sweden; The Australian National University, AUSTRALIA

## Abstract

To establish strategic priorities for the Public Health Agency of Sweden we prioritized pathogens according to their public health relevance in Sweden in order to guide resource allocation. We then compared the outcome to ongoing surveillance. We used a modified prioritization method developed at the Robert Koch Institute in Germany. In a Delphi process experts scored pathogens according to ten variables. We ranked the pathogens according to the total score and divided them into four priority groups. We then compared the priority groups to self-reported time spent on surveillance by epidemiologists and ongoing programmes for surveillance through mandatory and/or voluntary notifications and for surveillance of typing results. 106 pathogens were scored. The result of the prioritization process was similar to the outcome of the prioritization in Germany. Common pathogens such as calicivirus and Influenza virus as well as blood-borne pathogens such as human immunodeficiency virus, hepatitis B and C virus, gastro-intestinal infections such as *Campylobacter* and *Salmonella* and vector-borne pathogens such as *Borrelia* were all in the highest priority group. 63% of time spent by epidemiologists on surveillance was spent on pathogens in the highest priority group and all pathogens in the highest priority group, except for *Borrelia* and varicella-zoster virus, were under surveillance through notifications. Ten pathogens in the highest priority group (*Borrelia*, calicivirus, *Campylobacter*, *Echinococcus multilocularis*, hepatitis C virus, HIV, respiratory syncytial virus, SARS- and MERS coronavirus, tick-borne encephalitis virus and varicella-zoster virus) did not have any surveillance of typing results. We will evaluate the possibilities of surveillance for the pathogens in the highest priority group where we currently do not have any ongoing surveillance and evaluate the need of surveillance for the pathogens from the low priority group where there is ongoing surveillance in order to focus our work on the pathogens with the highest relevance.

## Introduction

National public health agencies need to prioritize their activities as there are limited resources for surveillance of on communicable diseases. Established routines, personal interests and short-term political agendas can lead to a situation where resources are spent on surveillance of pathogens with less relevance for public health. The Public Health Agency of Sweden identified the need to use a structured method that takes relevant aspects into account in order to rationally prioritize between different pathogens when allocating resources for surveillance.

In the literature we found several attempts to create frameworks and procedures for prioritization in public health [1–5]. Some attempts have also been made to prioritize among pathogens causing communicable diseases [6, 7]. The method we identified to be most appropriate to our needs was one that was developed at the Robert Koch Institute in Germany in 2011 [8–10].

The Robert Koch Institute started by generating a list of pathogens to prioritize and included pathogens that were a) notifiable according to German law, b) reportable within the European Union, c) reportable to the World Health Organization under the International Health Regulations, d) agents with potential for deliberate release, and e) pathogens represented in the “Control of Communicable Disease Manual” by Heymann et al. [11] occurring in Germany. Some pathogens were then grouped together when biologically and clinically plausible.

The Robert Koch Institute invited ten senior external experts and ten internal experts and asked them to score the pathogens with -1, 0 or 1 for ten variables“Incidence”, “Work and school absenteeism”, “Health care utilization”, “Chronicity of illness or sequelae”, “Case fatality rate”, “Proportion of events requiring public health actions”, “Trend”, “Public attention”, “Prevention and Treatment possibilities”) (Table 1). The scoring was done in a modified Delphi process consisting of two rounds. First, the panel of experts received the list of pathogens they should score. They then gave their score and motivation for the score given. The scores and motivations were anonymized and sent out in a second round so each participant could change their answers in light of the other experts’ replies. This was done so that the panel could reach a consensus without letting “group dynamics” affect the process.

**Table 1:** Prioritization variables. The ten variables that pathogens were scored for during the prioritization and the criteria for each score.

No.	Criteria	Scoring values		
		-1	0	1
1	Incidence (including illness, symptomatic infections, asymptomatic infections but not carriership or normal flora)	<1/100 000	1-20/100 000	>20/100 000
2	Work and school absenteeism*	This pathogen causes negligible proportion of absenteeism due to an infectious illness	This pathogen causes a small to moderate proportion of absenteeism due to an infectious illness	This pathogen causes a larger proportion of absenteeism due to an infectious illness
3	Health care utilization (primary health care and hosptitalization)	This pathogen causes a negligligle proportion of health care utilization due to an infectious illness	This pathogen causes a small to moderate proportion of health care utilization due to an infectious illness	This pathogen causes a large proportion of health care utilization due to an infectious illness
4	Chronicity of illness or sequelae*	This pathogen causes a negligible amount of chronicity or persistent sequelae	This pathogen causes a moderate amount of chronicity or persistent sequelae	This pathogen causes a large amount of chronicity or persistent sequelae
5	Case fatality rate**	<0.01%	0.01%-1%	>1%
6	Proportion of events requiring public health actions (see Note 2 for explanation)**	A small proportion of the estimated total number of events or exceptional events require public health actions (<25%)	A moderate proportion of the estimated total number of events or exceptional events require public health actions (25–75%)	A large proportion of the estimated total number of events or exceptional events require public health actions (>75%)
7	Trend	Diminishing incidence rates	Stable incidence rates	Increasing incidence rates
8	Public attention (including political agenda and public perception)*	Risk perception of this pathogen by the gernal public is low and it is not on the political agenda	Risk perception of this pathogen by the gernal public is moderate and informal political expectations/agenda is present	This pathogen implies international duties or its risk perception by the general public is high or it is explicitly high on political agenda
9	Prevention possibilites and needs (including vaccines)**	Preventive potential seems low or the disease does not require prevention or effective prevention strategies are well-established; no need for significant strategy modification	Measures for prevention are established but there is need to improve their effctiveness	Need for prevention is established but currently no effective preventive measures are available
10	Treatment possibilities and needs (including AMR)	Medical treatment is rarely necessary or effective regimens are well-established; no need for significant modifications	Medical treatme regimens are established but there is need to improve their effectiveness	Need for medical treatment is established but currently no effective treatment is available or AMR limits treatment options

AMR = anitimicrobial resistance

Note 1. All criteria apply to the geographical settings where the prioritization is conducted; the time-frame applicable to the requested epidmiological data should be defined prior to the process initiation and depends on the frequency with which pathogens are planned to be re-scored. Indicated numercial thresholds apply to the country where the prioritization is conducted: In other geographical settings different thresholds might need to be considered.

Note 2. Event is defined as the occurrence of a disease that is unusual with respect to a particular time, place or circumstances. For certain infectious diseases one case may be sufficient to constitute an event (e.g. polio virus). Public health actions are any kind of targeted actions aiming to identify the nature of the event and/or to apply control measures in response to the event occurrence.

*assessed against the total burden of infectious diseases.

**assessed for each particular pathogen in question, e.g., the criterion "Treatment possibilities and needs" refers to availability and adequacy of treatment for each case of an illness caused by a particular pathogen but does not take into account the incidence of illnesses or the availability of preventive measures.

Internal and external experts were then asked to give a weight of 0 to 10 for each criterion and the median value was then used to give each criterion a weight. The score for each pathogen was multiplied by the weight and re-scaled from 0–100. The pathogens were divided into four priority groups (the highest, high, medium and low) based on their final score. The cut-off limits for the groups were 76–100, 51–75, 26–50, and 0–25.

We made some modifications to the prioritization method and then applied it in Sweden in order to generate a prioritized list of pathogens. We then measured the current allocation of resources for surveillance at the Public Health Agency of Sweden in order to examine to what degree resources were spent after public health relevance.

## Methods

We used the methodology developed at the Robert Koch Institute [8], with some modifications as described below.

### Selection of pathogens

We started with the list of pathogens that had been used during the prioritization procedure in Germany. We then distributed the list among experts at the Public Health Agency of Sweden (at the time “The Swedish Institute for Communicable Disease Control”). Experts at each unit at the institute were allowed to make additions to the list if they deemed it to be necessary. After additions we then removed pathogens where spread is not ongoing, or deemed not likely to occur, in Sweden. This gave us the final list of pathogens to prioritize.

### Scoring

We used the same scoring criteria as in the original method with some modifications. The criterion for incidence was changed to include not only symptomatic infections but also asymptomatic infections (but not asymptomatic colonization with bacteria). We also changed the criteria of “chronicity of illness and sequelae” from the contribution of a pathogen to the total amount of chronicity or sequelae to “the risk for each pathogen to cause chronicity and sequelae” (Table 1).

### Delphi process

Five experts were invited to participate in the scoring procedure. One expert from the National Board of Health and Welfare, one from the Public Health Agency of Sweden and three experts who were County Communicable Disease Control Officers. We sent out the pathogens for scoring in batches of around ten pathogens in each batch.

### Weighting

The weighting step was removed. This was the other modification of the German method in addition to those mentioned in the “Scoring” paragraph.

### Ranking

As in the original method, the scores for each variable for each pathogen were summarized and the score for the pathogen was then re-scaled to 1–100 in order to reach the final score.

### Comparison of Swedish and German prioritization process

We then compared the outcome of the prioritization process in Sweden to the outcome in Germany by examining which (if any) pathogens were prioritized more than one group higher or lower in the Swedish list compared to the German list. For those pathogens we examined how the scores for each variable differed.

### Comparison of priority groups in relation to the current allocation of resources

In order to examine how resources are currently allocated in relation to the outcome of the prioritization procedure, we selected three key indicators. First, we compiled a list of all pathogens that are currently under surveillance by the Public Health Agency of Sweden through mandatory and/or voluntary notifications of cases by clinicians and/or laboratories. We did not include systems for “event-based surveillance”. Even if only one manifestation of the pathogen (e.g. invasive infection or strains with antibiotic resistance) was under surveillance, that pathogen was considered to be under surveillance. In addition, according to Swedish law, all cases of viral meningoencephalitis are notifiable. Thus we considered the viruses that can cause viral meningoencephalitis to be under surveillance. Second, we asked all epidemiologists responsible for surveillance of a particular pathogen at the National Public Health Agency to estimate how many full-time equivalents (FTEs) (1 year of full time work = 1 FTE) that were spent on surveillance activities for each pathogen, as an average over a year (if several people worked with one pathogen their work time was combined). Time spent on surveillance of typing results was not included in this estimation.

Third, we compiled a list of all pathogens that were under surveillance through typing (e.g. sequencing, pulsed-field gel electrophoresis and resistance patterns for antibiotics) of all isolates or a sample of isolates depending on the pathogen. Either through collection of typing results from the laboratories at the county level or where isolates were collected and typed at the Public Health Agency of Sweden.

### Ethics statement

This work was considered to be part of the duties of The Public Health Agency of Sweden and clearance was given by The Public Health Agency of Sweden before the study was started. The study did not involve human subjects. Experts in infectious diseases were consulted as part of their job as communicable disease officers.

## Results

### The prioritization in Sweden

Using the process described in the method section “Selection of pathogens” we generated a list of 106 pathogens. This list of 106 pathogens were distributed in batches to the experts for scoring. After scoring and re-scaling of the scores 21 pathogens were in the highest priority group, 28 in the high priority group, 37 in the medium priority group and 20 in the low priority group (**Table 2**and **S1 Table**).

**Table 2:** Prioritized pathogens. A list of pathogens in each priority group with information on type of ongoing surveillance (if any), Sweden, 2013. Group of priorizitation in Germany (8) is given in parenthesis.

1. Highest priority group (n = 21)	2. High priority group (n = 28)	3. Medium priority group (n = 37)	4. Low priority group (n = 20)
*Borrelia burgdorferi (3)*	*Bacillus anthracis (3) (M)*	*Acinetobacter spp*. (2)	*Mycobacterium* (non-*tuberculosis*) (3) (M)
Calicivirus (noro- and sapovirus) (2) (V)	*Bordetella pertussis (3) (M*, *TP)*	Adenovirus (2)	BK-virus (5)
Campylobacter spp (1) (M)	*Candida* spp. (3) (M)	*Aspergillus* spp. (2)	Cercarial dermatitis (5)
*Clostridium difficile (1) (V*, *TP*, *TC)*	*Chlamydia trachomatis (1) (M)*	*Brucella* spp. (2) (M)	*Chlamydophila psittaci (4) (M)*
*Escherichia coli* (non-gastro illnesses) including ESBL (1) (M, TP, TC)	*Citrobacter* spp. incl. ESBL (3) (M, TC)	*Burkholderia cepacia (3)*	*Clostridium perfringens (3)*
*Echinococcus multilocularis (5) (M)*	*Corynebacterium diphtheriae (2) (M)*	*Chlamydia pneumoniae* / *Chlamydophila pneumoniae (4)*	*Cryptococcus neoformans* and *gattii (3)*
*Escherichia coli* (shiga toxin producing i.e. EHEC) (1) (M,TP, TC)	*Cryptosporidium parvum/hominis (2) (M)*	*Clostridium botulinum (3) (M)*	*Entamoeba histolytica (4) (M)*
Hepatitis B virus (1) (M, TC)	*Enterobacter* spp. Incl. ESBL (1) (M, TC)	*Clostridium tetani (3) (M)*	Helminths (tapeworms)**** (4)
Hepatitis C virus (1) (M)	*Enterococcus* spp. (blood) incl. Vancomycin resistant (VRE) (1) M, TP, TC)	Coronaviruses (3)	Helminths (flukes)** (4)
Human papilloma virus (HPV) (2) (V, TP)	Epstein-Barr virus (HHV-4) (2)	*Corynebacterium ulcerans* and *Corynebacterium pseudotuberculosis* (2)	Helminths (nematodes)*** (4)
Human immunodeficiency virus (HIV) (1) (M)	*Giardia lamblia (2) (M)*	*Coxiella burnetii (3) (M)*	Hepatitis E virus (2) (M)
Influenza virus (1) (M, V, TP)	*Haemophilus influenzae (2) (M*, *TP*, *TC)*	Cytomegalovirus (HHV-5) (3)	HHV-8 (Kaposi's sarcoma associated) (3)
Measels virus (1) (M, TP)	*Helicobacter pylori (1)*	*Echinococcus granulosis (5) (M)*	*Histoplasma capsulatum (4)*
*Neisseria meningitidis (1) (M*, *TP)*	Klebsiella spp incl. ESBL (1) (M, TP, TC)	*Enterobius vermicularis (5)*	JC-virus (5) (M)
Respiratory syncytial virus (RSV) (1) (V)	*Legionella pneumophila (1) (M)*	Enteroviruses spp. incl. echovirus and Coxsackievirus (2) (M, TP)	Molluscipoxvirus (4)
*Salmonella* spp. (non-*T*yphi and non-Paratyphi) (1) (M, TP)	*Listeria monocytogenes (2) (M*, *TP)*	*Francisella tularensis (3) (M)*	Sindbisvirus (5)
SARS- and MERS coronavirus (2) (M)	*Mycobacterium tuberculosis (1) (M*, *TP)*	Hepatitis A virus (2) (M, TP)	Parvovirus B19 (3)
*Staphylococcus aureus* incl. methicillin resistent (MRSA) and *Staphylococcus aureus*, toxogenic (1) (M, TP, TC)	*Mycoplasma* spp. (2)	Hepatitis D virus (2) (M)	*Salmonella* Typhi and *Salmonella* Paratyphi *(3) (M*, *TP)*
*Streptococcus pneumoniae (1) (M*, *TP)*	*Naegleria fowleri (5)*	*Herpes simplex virus* (HSV)-1 (3) (M)	Vibrio (non-cholerae): *V*. *parahaemolyticus*, *V*. *vulnificus* and *V*. *cholerae* (non O1 and O139) (3) (M)
Tick borne encephalitis virus (2) (M)	*Neisseria gonorrhoeae (2)*	*Herpes simplex virus* (HSV)-2 (3) (M)	*Vibrio cholerae (3) (M)*
Varicella zoster virus (1)	Mumps virus (2) (M, TP)	Humant T-cell lymphotrophic virus (HTLV) (3) (M)	
	Pediculosis (head, body and pubic lice) (2)	Unidentified agent causing Kawasaki syndrome (4)	
	*Pseudomonas* spp. (1) (TC)	*Leptospira interrogans (3) (M)*	
	Puumalavirus (1*****) (M)	Metapneumovirus (5)	
	Rabies virus (2) (M)	Mucorales (Zygomycetes) (4)*	
	Rota virus (2) (TP)	Parainfluenza virus (2)	
	*Shigella* spp. (3) (M, TP)	*Pneumocyctis jiroveci (4)*	
	*Streptococcus* spp other than *Streptococcus pneumoniae (1) (M*, *TP*, *TC)*	Rhinoviruses (3)	
		Rubellavirus (3) (M,TP)	
		*Sarcoptes scabiei (3)*	
		*Staphylococcus epidermidis/Coagnulase-negative staphylococci (1)*	
		*Stenotrophomonas (Pseudomonas) maltophilia (3)*	
		*Toxoplasma gondii (2) (M)*	
		*Treponema pallidum (3) (M)*	
		*Trichinella spiralis (3) (M)*	
		*Trichophyton* spp, *Microsporum* spp and *Epidermophyton* spp.(dermatophytes) (5)	
		*Yersinia enterocolitica* and *pseudotuberculosis (2) (M)*	

* In Germany included in Fungi (other)

**Helminths (flukes) group includes: Clonorchis sinensis, Opisthorchis felineus, Opisthorchis viverrini, Fasciolopsis buski, Fasciolopsis gigantica and Fasciolopsis hepatica, Paragonimius, Schistosoma

***Helminths (nematodes) group includes: Ancylostoma braziliense and caninum, Angiostrongylus, Ascaris lumbricoides, Capillaria philippinensis, hepatica and aerophila, Dracunluse meditensis, Enterobius vermicularis, Filaria (Onchocerca volvulus, Loa loa, Wuchereia bancrofti, Brugia malayi and Brugia timori). Hookworms (Ancylostoma duodenale and Necator americanus), Strongyloides stercoralis, Toxocara canis and cati, Trichuris trichiura. Trichinella spp. was scored as a separate pathogen

****Helminths (tapeworms) group includes: Diphyllobotrium latum, Echinococcus granulosus, Echinococcus multilocularis, Hymenolepsis nana, Taenia saginata, Taeniasolium

*****In Germany belonging to Hantavirus group

M = Mandatory notifiable, V = Voluntary notifiable, TP = Typing at the Public Health Agency of Sweden, TC = Surveillance of typing results done at county level

### Comparison of the outcome of the Swedish and the German prioritization process

In Sweden 106 pathogens were prioritized compared to 127 in Germany. Twelve pathogens were prioritized in Sweden but not in Germany and 31 pathogens were prioritized in Germany but not in Sweden.

Three pathogens differed by more than one group when comparing the Swedish to the German list (**Table 2)**. *Borrelia* was placed in the highest priority group in Sweden but in the medium priority group in Germany. *Staphylococcus epidermidis* (coagulase-negative *Staphylococcus*) was placed in the medium priority group in Sweden but in the highest priority group in Germany. Hepatitis E virus was placed in the lowest priority group in Sweden but in the high priority group in Germany. *Borrelia* differed in the scores for “Chronicity of illness or sequelae”, *S*. *epidermidis* differed in both “Incidence” and “Chronicity of illness or sequelae” and hepatitis E differed in “Incidence” when comparing the Swedish to the German scoring for each variable (data not shown).

### Comparison of priority groups in relation to ongoing surveillance

At the National Public Health Agency of Sweden we currently conduct surveillance based on mandatory notifications for 59 of the pathogens and on voluntary notifications for 5 pathogens, of the 106 pathogens that were prioritized (**Table 2**).

Of the 63 pathogens under surveillance through notification of cases 19 (30%) were in the highest priority group, 19 (30%) in the high priority group, 17 (27%) in the medium priority group and 8 (13%) in the low priority group (**Table 2**). Two pathogens, (*Borrelia* and varicella-zoster virus) were in the highest priority group but did not have any ongoing surveillance through notification of cases. Eight pathogens under surveillance through notifications of cases were placed in the low priority group (atypical mycobacteria, *Chlamydiophila psittacci*, *Entamoeba histolytica*, hepatitis E virus, JC-virus, *Salmonella* Typhi and Paratyphi, *Vibrio cholerae* and *Vibrio* non-*cholerae*).

Work-time for epidemiologist for surveillance corresponded to 7.0 FTE per year (this was however distributed among more epidemiologist than 7 since some work part-time and for some not all of their work time is dedicated to surveillance related activities) (**S2 Table**). 4.5 FTE:s (63%) of the FTEs were spent on pathogens in the highest priority group. 2.2 FTE:s (31%) were spent on pathogens in the high priority group and 0.3 FTE:s (4%) were spent on pathogens in the medium priority group.

Of the 28 pathogens under surveillance through typing 11 (39%) where in the highest priority group, 13 (46%) were in the high priority group, 3 (11%) in the medium priority group and 1 (4%) were in the low priority group (**Table 2**). Ten pathogens in the highest priority group did not have any surveillance through typing (*Borrelia*, Campylobacter, calicivirus, *E*. *multilocularis*, hepatitis C virus, human immunodeficiency virus, Respiratory syncytial virus, SARS- and MERS coronavirus, tick borne encephalitis virus and varicella-zoster virus). In the low priority group there was only surveillance through typing for *Salmonella* Typhi *and* Paratyphi.

## Discussion

We used a standardized procedure developed at the Robert Koch Institute to generate a list of pathogens prioritized for surveillance to be used by the Public Health Agency of Sweden.

Before applying the Robert Koch Institute prioritization procedure in Sweden we made two modifications in order to make the procedure less time consuming. First, we excluded pathogens where there was no ongoing spread in Sweden or where spread in Sweden was not deemed likely. Second, we excluded the weighting step described in the original procedure. The reasons for excluding the weighting step was that in the experience from Germany for some variables, such as “Public attention” there was a high variation in the weight given by different experts. In addition for some variables the weight given differed between different groups of experts in Germany. For example “Incidence” was given a high weight by epidemiologists and public health experts but a low weight by clinicians. The relationship was reversed for the criteria “Work and school absenteeism” and “Health care utilization”. The number of experts invited from each category therefore affected the weighting score. Due to these limitations and in order to make the procedure less time consuming we decided to remove the weighting step.

We also made modifications to the scoring criteria for two of the ten variables. We changed the criteria for “incidence” so that it would also include asymptomatic infections. We made this change since even asymptomatic infections can require interventions such as contact tracing and vaccination of those exposed. In addition, the Swedish sureveillance system for notifiable diseases does not include data on whether the infection was symptomatic or not. Thus, there is a lack of epidemiological data in Sweden that differentiates between symptomatic and asymptomatic infections. We also modified the criteria for chronicity of illness and sequelae from the contribution of a pathogen to the total amount of chronicity or sequelae for all pathogens combined to the individual risk for each pathogen to cause chronicity and sequalae. This was done in order to give the incidence of a pathogen less influence as incidence was already included as a separate variable.

Only three of the pathogens (*Borrelia*, *S*. *epidermidis* and hepatitis E virus) that were included in the prioritization procedure in both Sweden and Germany differed by more than one priority group. To some extent this difference could have been due to changes in the scoring criteria for the variables “Incidence” and for “Chronicity of illness or sequelae”, but this was likely not the entire explanation to the differences in priority groups for these pathogens. True differences in incidence could explain why “Work and school abseentism” and “Health care utilization” was scored lower in Sweden than in Germany for *S*. *epidermidis*. The incidence for *Borrelia* could also be higher in Sweden which could have affected the scoring for “Incidence” and “Chronicity of illness or sequelae”. Public and media attention probably also differ between countries, which could explain why “Public attention” for *S*. *epidermidis* and hepatitis E scored lower in Sweden than in Germany. Lower scores for “Prevention possibilities and needs” and “Treatment possibilities and needs” for hepatitis E in Sweden than in Germany more likely reflect a difference in the interpretation of the available evidence on if and how this infection should be treated than a real difference in the available interventions for prevention or treatment between Sweden and Germany.

We compared the ongoing surveillance at the Public Health Agency of Sweden to the prioritized list of pathogens which gave a useful indication on how well resources were currently allocated.

When comparing the existing surveillance through notifications in Sweden to the priority groups we found that the Public Health Agency of Sweden did not have any surveillance through notifications for two pathogens in the highest priority groups (*Borrelia* and varicella-zoster virus).

We found that for eight pathogens in the low priority group (*Mycobacterium* non-*tuberculosis*, *Chlamydophila psittaci*, *Entamoeba histolytica*, hepatitis E virus, JC-virus, *Salmonella* Typhi and Paratyphi, *Vibrio cholerae* and *Vibrio* non-*cholerae*) there was ongoing surveillance through notifications. This could partly be explained by the law requiring all viral meningoencephalitis to be under surveillance, thus JC virus is under surveillance. For *Salmonella* our method divided *Salmonella* spp and *Salmonella* Typhi and non-Typhi but the law requires surveillance for all forms of *Salmonella* which could explain why *Salmonella* Typhi and non-Typhi was under surveillance although it was considered to be of low relevance to public health.

When we estimated FTEs for surveillance through notifications per pathogen we found that 95% of FTEs were spent on surveillance through notifications for pathogens in the highest and the high priority group. We take this as an indication that surveillance through notifications at the Public Health Agency of Sweden is already focused on the most important pathogens.

We also compared the existing surveillance through typing results in Sweden to the different priority groups. The surveillance through typing was to a large extent focused on the highest and the high priority group. However, ten pathogens in the highest priority group lacked programs for surveillance through typing (*Borrelia*, *Campylobacter*, calicivirus, *E*. *multilocularis*, hepatitis C, human immunodeficiency virus, respiratory syncytial virus, SARS- and MERS coronavirus, tick borne encephalitis virus and varicella-zoster virus). Reasons for this could be that in the past, suitable methods for typing for some of the pathogens were lacking, but as new techniques such as whole-genome sequencing become less expensive, this might change. Another reason for lack of surveillance through typing be due to the pathogenesis of the disease caused by the pathogen under surveillance. For example, when the symptoms for tick-borne encephalitis virus are seen, the virus is usually no longer detectable and isolation for typing is therefore not possible.

Discrepancies between ongoing surveillance through notifications and surveillance through typing and the outcome of the prioritization procedure should be interpreted with caution since there might be good reasons for not having surveillance for the pathogens in the highest priority group or for having surveillance for pathogens in the low priority group. The results should mainly function as an indication that the need for surveillance, or lack of surveillance, for certain pathogens should be evaluated. A key aspect to evaluate would be the availability of actions that could be triggered by the data from the surveillance system.

This study had limitations. Despite the modifications to the procedure, the Delphi process was very time consuming and took over a year to carry out. Apart from the associated costs, it could have led to “expert fatigue” and a changing interpretation of the ranking criteria over time. Another limitation of this study is that we estimated full-time equivalents for surveillance through notifications per pathogen by self-assessment. This might not accurately reflect the actual time spent on surveillance through notifications for over a year. For example surveillance activities with a yearly variation (e.g. for influenza virus) might be overestimated if the assessment takes place during the influenza season and vice versa. In addition to that, FTEs per pathogen also depend on the efficiency of the surveillance system. A poorly designed surveillance system might require more time spent than several well designed systems. We did not have the possibility to assess FTEs for surveillance of typing results. Furthermore, we did not measure the resources spent on research activities at the Public Health Agency of Sweden. It would be of interest to find out how they are distributed among the priority groups.

## Conclusions

In conclusion we have generated a list of pathogens prioritized for surveillance that can be used by the Public Health Agency of Sweden. The list could also be used when prioritizing among public health research projects on communicable diseases. We have made the prioritization method less time consuming when comparing the prioritized list to the current activities at the Public Health Agency of Sweden we found that to a large extent our activities are already focused on the pathogens with higher priority. We did however identify pathogens where surveillance (or discontinuation of surveillance) should be evaluated.

The Public Health Agency of Sweden will consider surveillance for the pathogens in the highest priority group where we currently do not have any ongoing surveillance and to evaluate the possibilities to stop the surveillance for the pathogens from the low priority group where there is ongoing surveillance. In addition the results indicate that other countries with a similar panorama of communicable diseases and the same level of health care as in Sweden and Germany could consider using the list from either Sweden or Germany in order to save time. For countries who plan to conduct the prioritization method we recommend to consider additional modifications of the prioritization method in order to make it less time consuming, such as replacing the Delphi procedure with a “mini-Delphi” that can take place during a one-day workshop, which has previously been done in other settings [12].

## Supporting Information

S1 Table Scores for each pathogen and all variables. Individual score, total score, re-scaled score and priority group for all pathogens prioritized. Sweden, 2013.
(DOCX)

S2 Table Full-time equivalents spent on surveillance for each pathogen. Self-estimated time spent on surveillance of notifications at The Public Health Agency of Sweden for all pathogens that were prioritized. Sweden, 2013.
(DOCX)
