# Correction: Communicable Diseases Prioritized According to Their Public Health Relevance, Sweden, 2013

**DOI:** 10.1371/journal.pone.0174491

**Published:** 2017-03-16

**Authors:** Viktor Dahl, Anders Tegnell, Anders Wallensten

There is an error in S1 Table. The first column of the eleventh row is incorrect. It should read: *Salmonella spp (non-Typhi and non Paratyphi)*. Please view the correct S1 Table below.

## Supporting information

S1 Table Scores for each pathogen and all variables. Individual score, total score, re-scaled score and priority group for all pathogens prioritized. Sweden, 2013.
(DOCX)
