# Supplementary material for: Communicable Diseases Prioritized According to Their Public Health Relevance, Sweden, 2013
Source: PLoS One. 2015 Sep 23;10(9):e0136353. doi: 10.1371/journal.pone.0136353 (PMC4580468; doi:10.1371/journal.pone.0136353)
Supplement: S2 Table — Self-estimated time spent on surveillance of notifications at The Public Health Agency of Sweden for all pathogens that were prioritized. Sweden, 2013. (DOCX) [file pone.0136353.s002.docx]

**S2 Table. Full-time equivalents spent on surveillance for each pathogen.** Self-estimated time spent on surveillance of notifications at The Public Health Agency of Sweden for all pathogens that were prioritized. Sweden, 2013.

| **Pathogen** | **Full time equivalents** |
| --- | --- |
| *Borrelia burgdorferi* |  |
| Calicivirus (noro- and sapovirus) | 0.2 |
| *Campylobacter* spp | 0.1 |
| *Clostridium difficile* | 0.15 |
| *Escherichia coli* (non-gastro illnesses) including ESBL | 0.16 |
| *Echinococcus multilocularis* |  |
| *Escherichia coli* (shiga toxin producing i.e. EHEC) | 0.5 |
| Hepatitis B virus | 0.25 |
| Hepatitis C virus | 0.25 |
| Human papilloma virus (HPV) |  |
| Human immunodeficiency virus (HIV) | 0.5 |
| Influenza virus | 0.75 |
| Measels virus |  |
| *Neisseria meningitidis* |  |
| Respiratory syncytial virus (RSV) | 0.15 |
| *Salmonella* spp. (non-Typhi and non-Paratyph*i*) |  |
| SARS- and MERS coronavirus |  |
| *Staphylococcus aureus* incl. methicillin resistent (MRSA) an*d Staphylococcus aureus* toxogenic | 0.7 |
| *Streptococcus pneumoniae* | 0.05 |
| Tick borne encephalitis virus | 0.3 |
| Varicella zoster virus |  |
| *Bacillus anthracis* |  |
| *Bordetella pertussis* |  |
| *Candida* spp. |  |
| *Chlamydia trachomatis* |  |
| *Citrobacter* spp. incl. ESBL | 0.16 |
| *Corynebacterium diphtheriae* |  |
| *Cryptosporidium parvum/hominis* | 0.25 |
| *Enterobacter* spp. including ESBL | 0.16 |
| *Enterococcus* spp. (blood) inc.l vancomycin resistant (VRE) | 0.3 |
| Epstein-Barr virus (HHV-4) |  |
| *Giardia lamblia* |  |
| *Haemophilus influenzae* |  |
| *Helicobacter pylori* |  |
| *Klebsiella* spp incl. ESBL | 0.16 |
| *Legionella pneumophila* | 0.5 |
| *Listeria monocytogenes* | 0.1 |
| *Mycobacterium tuberculosis* | 0.25 |
| *Mycoplasma* spp. |  |
| *Naegleria fowleri* |  |
| *Neisseria gonorrhoeae* |  |
| Mumps virus |  |
| Pediculosis (head. body and pubic lice) |  |
| *Pseudomonas* ssp. |  |
| Puumalavirus | 0.15 |
| Rabies virus |  |
| Rota virus |  |
| *Shigella* spp. | 0.15 |
| *Streptococcus spp* other than *Streptococcus pneumoniae* | 0.05 |
| Acinetobacter |  |
| Adenovirus |  |
| *Aspergillus* spp. |  |
| *Brucella* spp. |  |
| *Burkholderia cepacia* |  |
| *Chlamydia pneumoniae* |  |
| *Clostridium botulinum* |  |
| *Clostridium tetani* |  |
| SARS- and MERS coronaviruses |  |
| *Corynebacterium ulcerans* and *Corynebacterium pseudotuberculosis* |  |
| *Coxiella burnetii* |  |
| Cytomegalovirus (HHV-5) |  |
| *Echinococcus granulosis* |  |
| *Enterobius vermicularis* |  |
| Enteroviruses incl. echovirus and Coxsackievirus |  |
| *Francisella tularensis* | 0.1 |
| Hepatitis A virus | 0.2 |
| Hepatitis D virus |  |
| *Herpes simplex virus* (HSV)-1 |  |
| *Herpes simplex virus* (HSV)-2 |  |
| Humant T-cell lymphotrophic virus (HTLV) |  |
| Unidentified agent causing Kawasaki syndrome |  |
| *Leptospira interrogans* |  |
| Metapneumovirus (paramyxo) |  |
| Mucorales (Zygomycetes) |  |
| Parainfluenza virus |  |
| *Pneumocyctis jiroveci* |  |
| Rhinoviruses |  |
| Rubellavirus |  |
| *Sarcoptes scabiei* |  |
| *Staphylococcus epidermidis (*coagnulase-negative *staphylococci)* |  |
| *Stenotrophomonas (Pseudomonas) maltophilia* |  |
| *Toxoplasma gondii* |  |
| *Treponema pallidum* |  |
| *Trichinella spiralis* |  |
| *Trichophyton* spp. *Microsporum* spp and *Epidermophyton* spp. (dermatophytes) |  |
| *Yersinia enterocolitica* and *pseudotuberculosis* |  |
| *Mycobacterium* other (non-*tuberculosis*) |  |
| BK-virus |  |
| Cerkariedermatitis |  |
| *Chlamydophila psittaci* |  |
| *Clostridium perfringens* |  |
| *Cryptococcus neoformans* and *gattii* |  |
| *Diphyllobotrium* spp. |  |
| *Entamoeba histolytica* | 0.01 |
| Helminths (tapeworms) |  |
| Helminths (flukes) |  |
| Helminths (nematodes) |  |
| Hepatitis E virus | 0.01 |
| HHV-8 (Kaposi's sarcoma associated) |  |
| *Histoplasma capsulatum* |  |
| JC-virus |  |
| Molluscipoxvirus |  |
| Sindbisvirus |  |
| Parvovirus B19 |  |
| *Salmonella* Typhi and *Salmonella* Paratyphi | 0.4 |
| Vibrio (non-*cholerae*): *V. parahaemolyticus*. *V. vulnificus* and *V. cholerae* (non O1 and O139) |  |
| *Vibrio cholerae* |  |
| Arthropod-borne viral enchephalitides |  |
| Prions causing *Creutzfeldt Jakob Disease* |  |
| Crimean-Congo hemorrhagic fever virus |  |
| Dengue fever virus | 0.1 |
| Ebola and Marburg virus |  |
| Lassa virus |  |
| Microspora and trichophyton |  |
| Mumps virus |  |
| Polio virus |  |
| Variola virus |  |
| Viruses. others causing hemorrhagic fevers (Chikungunya. Rift Valley) |  |
| West Nile |  |
| Yellow fever virus |  |
| *Bacillus cereus* |  |
| *Bartonella quintana* |  |
| *Burkholderia mallei* and *pseudomallei* |  |
| *Escherichia coli* enteropathogenic (non STEC/HUS) |  |
| *Leishmania* spp. |  |
| *Plasmodium* spp. | 0.05 |
| *Rickettsia prowazekii* and *Orientia tsutsugamushi* |  |
| *Rickettsia* spp. |  |
| *Trichomonias vaginalis* |  |
| *Trypanosoma brucei gambiensi* and *brucei rhodesiensi* |  |
| Vaccinia virus |  |
| *Yersinia pestis* |  |
| Actinomycosis |  |
| Astro virus |  |
| *Cyklospora cayetanensis* |  |
| Fungi (other) |  |
