# Supplementary material for: Correction: Communicable Diseases Prioritized According to Their Public Health Relevance, Sweden, 2013
Source: PLoS One. 2017 Mar 16;12(3):e0174491. doi: 10.1371/journal.pone.0174491 (PMC5354440; doi:10.1371/journal.pone.0174491)
Supplement: S1 Table — Individual score, total score, re-scaled score and priority group for all pathogens prioritized. Sweden, 2013. (DOCX) [file pone.0174491.s001.docx]

**S1 Table. Scores for each pathogen and all variables.** Individual score, total score, re-scaled score and priority group for all pathogens prioritized. Sweden, 2013.

| **Pathogen** | **Incid-ence** | **Absent-eeism** | **Health care utilization** | **Chronicity of illness or sequelae** | **Case fatality rate** | **Public health actions** | **Trend** | **Public attention** | **Prevention possibilites and needs** | **Treatment possibilities and needs** | **Total score** | **Re-scaled score** | **Prio group** |
| --- | --- | --- | --- | --- | --- | --- | --- | --- | --- | --- | --- | --- | --- |
| Influenza virus | 1 | 1 | 1 | -1 | 0 | 0 | 0 | 1 | 1 | 0 | 4 | 100 | 1 |
| Respiratory syncytial virus (RSV) | 1 | 1 | 1 | 0 | -1 | 0 | 0 | 0 | 1 | 1 | 4 | 100 | 1 |
| Calicivirus | 1 | 1 | 1 | -1 | 0 | 0 | 1 | 1 | 1 | -1 | 4 | 100 | 1 |
| Tick borne encephalitis virus | 0 | 0 | 0 | 1 | 0 | 0 | 1 | 1 | 0 | 1 | 4 | 100 | 1 |
| Human immunodeficiency virus (HIV) | 0 | 0 | 0 | 1 | 0 | 1 | 0 | 1 | 0 | 0 | 3 | 92 | 1 |
| *Streptococcus pneumoniae* | 1 | 1 | 1 | 0 | 0 | -1 | 0 | 0 | 0 | 1 | 3 | 92 | 1 |
| Hepatitis B virus | 0 | 0 | 0 | 1 | 0 | 1 | 0 | 0 | 0 | 0 | 2 | 85 | 1 |
| Hepatitis C virus | 1 | 0 | 0 | 0 | 0 | 1 | 0 | -1 | 1 | 0 | 2 | 85 | 1 |
| *Escherichia coli*  (shiga toxin producing i.e. EHEC) | 0 | 0 | 0 | 0 | 0 | 1 | 1 | 0 | 0 | 0 | 2 | 85 | 1 |
| *Salmonella spp (non-Typhi and non Paratyphi)* | 1 | 0 | 0 | 0 | 0 | 1 | -1 | 1 | 0 | 0 | 2 | 85 | 1 |
| *Echinococcus multilocularis* | -1 | -1 | -1 | 1 | 1 | 1 | 0 | 1 | 0 | 1 | 2 | 85 | 1 |
| *Clostridium difficile* | 1 | 0 | 1 | 0 | 0 | 0 | 0 | 0 | 0 | 0 | 2 | 85 | 1 |
| *Staphylococcus aureus* incl. methicillin resistant (MRSA) | 1 | 1 | 1 | -1 | -1 | 0 | 0 | 0 | 0 | 1 | 2 | 85 | 1 |
| Human papilloma virus (HPV) | 1 | 0 | 1 | 1 | -1 | -1 | 0 | 1 | 0 | -1 | 1 | 77 | 1 |
| *Neisseria meningitidis* | -1 | -1 | -1 | 1 | 1 | 1 | 0 | 1 | 0 | 0 | 1 | 77 | 1 |
| Measels virus | -1 | -1 | -1 | 1 | 0 | 1 | 0 | 1 | 0 | 1 | 1 | 77 | 1 |
| Varicella-zoster virus | 1 | 1 | 0 | 1 | -1 | -1 | 0 | 0 | 0 | 0 | 1 | 77 | 1 |
| SARS and MERS coronavirus | -1 | -1 | -1 | 0 | 1 | 1 | 0 | 1 | 0 | 1 | 1 | 77 | 1 |
| *Campylobacter* spp | 1 | 0 | 0 | 0 | -1 | 0 | 1 | 0 | 0 | 0 | 1 | 77 | 1 |
| *Borrelia burgdorferi* | 1 | 0 | 1 | 0 | -1 | -1 | 0 | 1 | 1 | -1 | 1 | 77 | 1 |
| *Escherichia coli*  (non-gastro illnesses) incl. ESBL | 1 | 1 | 1 | -1 | -1 | 0 | 0 | 0 | -1 | 1 | 1 | 77 | 1 |
| *Chlamydia trachomatis* | 1 | -1 | 1 | 0 | -1 | 1 | 0 | 0 | 0 | -1 | 0 | 69 | 2 |
| *Mycobacterium tuberculosis* | 0 | -1 | -1 | -1 | 0 | 1 | 0 | 1 | 1 | 0 | 0 | 69 | 2 |
| Epstein-Barr virus (HHV-4) | 1 | 0 | 0 | 0 | -1 | -1 | 0 | 0 | 1 | 0 | 0 | 69 | 2 |
| *Shigella* spp. | 0 | 0 | 0 | 0 | 0 | 1 | 0 | -1 | 0 | 0 | 0 | 69 | 2 |
| Rota virus | 1 | 1 | 1 | -1 | -1 | -1 | 0 | 0 | 1 | -1 | 0 | 69 | 2 |
| Rabies virus | -1 | -1 | -1 | -1 | 1 | 1 | 0 | 1 | 0 | 1 | 0 | 69 | 2 |
| *Helicobacter pylori* | 1 | -1 | 1 | 0 | -1 | -1 | 0 | 0 | 1 | 0 | 0 | 69 | 2 |
| *Klebsiella* spp incl. ESBL | 1 | 0 | 1 | -1 | -1 | 0 | 0 | 0 | -1 | 1 | 0 | 69 | 2 |
| *Streptococcus* spp other than *Streptococcus pneumoniae* | 1 | 1 | 1 | -1 | -1 | 0 | 0 | 0 | 0 | -1 | 0 | 69 | 2 |
| *Neisseria gonorrhoeae* | 0 | -1 | -1 | 0 | -1 | 1 | 1 | -1 | 0 | 1 | -1 | 62 | 2 |
| *Corynebacterium diphtheriae* | -1 | -1 | -1 | -1 | 1 | 1 | 0 | 1 | 0 | 0 | -1 | 62 | 2 |
| *Haemophilus influenzae* | 0 | 0 | -1 | 1 | 1 | -1 | 0 | 0 | 0 | -1 | -1 | 62 | 2 |
| *Legionella pneumophila* | 0 | -1 | -1 | 0 | 1 | 1 | 0 | 0 | 0 | -1 | -1 | 62 | 2 |
| *Candida* spp. | 1 | -1 | 1 | -1 | -1 | -1 | 1 | 0 | 0 | 0 | -1 | 62 | 2 |
| Pediculosis | 1 | -1 | 0 | -1 | -1 | 0 | 1 | 0 | 0 | 0 | -1 | 62 | 2 |
| *Listeria monocytogenes* | 0 | -1 | -1 | 0 | 1 | 1 | 0 | 0 | 0 | -1 | -1 | 62 | 2 |
| Puumala virus | 0 | 0 | 0 | -1 | 0 | -1 | 0 | 0 | 0 | 1 | -1 | 62 | 2 |
| *Citrobacte*r spp. incl. ESBL | 0 | -1 | 0 | -1 | 0 | 0 | 1 | -1 | 0 | 1 | -1 | 62 | 2 |
| Enterococcus spp. (blood) incl. vancomycin resistant (VRE) | 0 | -1 | 0 | -1 | 0 | 0 | 0 | 0 | 0 | 1 | -1 | 62 | 2 |
| Hepatitis D virus | -1 | -1 | -1 | -1 | 1 | 1 | 0 | -1 | 0 | 1 | -2 | 54 | 2 |
| *Mycoplasma* spp. | 1 | 1 | 1 | -1 | 0 | -1 | 0 | -1 | -1 | -1 | -2 | 54 | 2 |
| *Bordetella pertussis* | 0 | 0 | 0 | -1 | 0 | 0 | -1 | 0 | 0 | 0 | -2 | 54 | 2 |
| Mumps virus | -1 | -1 | -1 | 0 | -1 | 1 | 0 | 0 | 0 | 1 | -2 | 54 | 2 |
| *Naegleria fowleri* | -1 | -1 | -1 | 0 | 1 | 1 | 0 | -1 | -1 | 1 | -2 | 54 | 2 |
| *Giardia lamblia* | 0 | 0 | 0 | 0 | -1 | 0 | 0 | -1 | 0 | 0 | -2 | 54 | 2 |
| *Cryptosporidium parvum* and *hominis* | 0 | 0 | -1 | 0 | -1 | 0 | 1 | -1 | 0 | 0 | -2 | 54 | 2 |
| *Bacillus anthracis* | -1 | -1 | -1 | -1 | 1 | 1 | 0 | 1 | 0 | -1 | -2 | 54 | 2 |
| *Pseudomonas* ssp. | 0 | -1 | 0 | -1 | 0 | 0 | 0 | -1 | 0 | 1 | -2 | 54 | 2 |
| *Enterobacter* spp. incl ESBL | 0 | -1 | 0 | -1 | 0 | 0 | 0 | -1 | 0 | 1 | -2 | 54 | 2 |
| *Treponema pallidum* | 0 | -1 | -1 | -1 | -1 | 1 | 0 | 1 | 0 | -1 | -3 | 46 | 3 |
| Human T-cell lymphotrophic virus (HTLV) | -1 | -1 | -1 | -1 | 0 | 1 | 0 | -1 | 0 | 1 | -3 | 46 | 3 |
| Herpes simplex virus (HSV)-2 | 1 | -1 | -1 | 1 | -1 | -1 | 0 | 0 | -1 | 0 | -3 | 46 | 3 |
| Parainfluenza virus | 1 | 1 | 1 | -1 | -1 | -1 | 0 | -1 | -1 | -1 | -3 | 46 | 3 |
| Rhino virus | 1 | 1 | 1 | -1 | -1 | -1 | 0 | -1 | -1 | -1 | -3 | 46 | 3 |
| *Pneumocyctis jiroveci* | 0 | -1 | 0 | -1 | 1 | -1 | 0 | -1 | 0 | 0 | -3 | 46 | 3 |
| Cytomegalovirus (HHV-5) | 1 | -1 | -1 | 0 | -1 | -1 | 0 | -1 | 1 | 0 | -3 | 46 | 3 |
| *Sarcoptes scabiei* | 0 | -1 | 0 | -1 | -1 | 0 | 0 | 0 | 0 | 0 | -3 | 46 | 3 |
| *Echinococcus granulosis* | -1 | -1 | -1 | 1 | 0 | 0 | 0 | 0 | -1 | 0 | -3 | 46 | 3 |
| *Acinetobacter* | -1 | -1 | -1 | -1 | 0 | 1 | 0 | -1 | 0 | 1 | -3 | 46 | 3 |
| *Burkholderia cepacia* | -1 | -1 | -1 | -1 | 0 | 1 | 0 | -1 | 0 | 1 | -3 | 46 | 3 |
| Staphylococcus epidermidis (coagnulase-negative staphylococci) | 0 | -1 | 0 | 0 | -1 | -1 | 0 | -1 | 0 | 1 | -3 | 46 | 3 |
| Herpes simplex virus (HSV)-1 | 1 | -1 | -1 | 1 | -1 | -1 | 0 | -1 | -1 | 0 | -4 | 38 | 3 |
| SARS- and MERS coronaviruses | 1 | 1 | 0 | -1 | -1 | -1 | 0 | -1 | -1 | -1 | -4 | 38 | 3 |
| *Aspergillus* spp. | -1 | -1 | -1 | -1 | 1 | -1 | 1 | -1 | 0 | 0 | -4 | 38 | 3 |
| *Brucella* spp | -1 | -1 | -1 | 0 | 0 | 1 | 0 | -1 | -1 | 0 | -4 | 38 | 3 |
| *Yersinia enterocolitica* and *pseudotuberculosis* | 0 | 0 | 0 | 0 | -1 | -1 | -1 | -1 | 0 | 0 | -4 | 38 | 3 |
| *Francisella tularensis* | 0 | 0 | 0 | -1 | -1 | 0 | 0 | -1 | 0 | -1 | -4 | 38 | 3 |
| *Clostridium botulinum* | -1 | -1 | -1 | 0 | 1 | 1 | 0 | -1 | -1 | -1 | -4 | 38 | 3 |
| *Coxiella burnetii* | -1 | -1 | -1 | 0 | 0 | 0 | 0 | -1 | 0 | 0 | -4 | 38 | 3 |
| *Trichinella spiralis* | -1 | -1 | -1 | 0 | 0 | 1 | 0 | 0 | -1 | -1 | -4 | 38 | 3 |
| *Trichophyton* spp, *Microsporum* spp and *Epidermophyton* spp. (dermatophytes) | 1 | -1 | 1 | 0 | -1 | -1 | 0 | -1 | -1 | -1 | -4 | 38 | 3 |
| *Stenotrophomonas* (*Pseudomonas*) *maltophilia* | -1 | -1 | -1 | -1 | 0 | 0 | 0 | -1 | 0 | 1 | -4 | 38 | 3 |
| Rubella virus | -1 | -1 | -1 | 0 | -1 | 1 | 0 | 0 | -1 | -1 | -5 | 31 | 3 |
| Metapneumo virus (paramyxo) | 1 | 0 | 0 | -1 | -1 | -1 | 0 | -1 | -1 | -1 | -5 | 31 | 3 |
| Adenovirus | 1 | 0 | 0 | -1 | -1 | -1 | 0 | -1 | -1 | -1 | -5 | 31 | 3 |
| *Corynebacterium ulcerans* and *Corynebacterium pseudotuberculosis* | -1 | -1 | -1 | -1 | 1 | 1 | 0 | -1 | -1 | -1 | -5 | 31 | 3 |
| *Clostridium tetani* | -1 | -1 | -1 | -1 | 1 | -1 | 0 | 0 | -1 | 0 | -5 | 31 | 3 |
| Unidentified agent causing Kawasaki syndrome | -1 | -1 | -1 | 1 | 0 | -1 | 0 | -1 | -1 | 0 | -5 | 31 | 3 |
| *Chlamydia pneumoniae* | 1 | 0 | 0 | -1 | -1 | -1 | 0 | -1 | -1 | -1 | -5 | 31 | 3 |
| Enterovirus spp. incl. echovirus and Coxsackievirus | 1 | -1 | 0 | -1 | -1 | -1 | 0 | 0 | -1 | -1 | -5 | 31 | 3 |
| *Toxoplasma gondii* | 1 | -1 | -1 | 0 | -1 | -1 | -1 | 0 | 0 | -1 | -5 | 31 | 3 |
| *Enterobius vermicularis* | 1 | -1 | -1 | -1 | -1 | -1 | 0 | 0 | 0 | -1 | -5 | 31 | 3 |
| Hepatitis A virus | -1 | -1 | -1 | -1 | -1 | 1 | 0 | 0 | 0 | -1 | -5 | 31 | 3 |
| *Leptospira interrogans* | -1 | -1 | -1 | 0 | 1 | 0 | 0 | -1 | -1 | -1 | -5 | 31 | 3 |
| Mucorales (Zygomycetes) | -1 | -1 | -1 | -1 | 1 | -1 | 1 | -1 | -1 | 0 | -5 | 31 | 3 |
| *Mycobacterium* other (non-*tuberculosis*) | 0 | -1 | -1 | 0 | -1 | -1 | 0 | -1 | -1 | 0 | -6 | 23 | 4 |
| *Clostridium perfringens* | -1 | -1 | -1 | -1 | -1 | 1 | 0 | 0 | -1 | -1 | -6 | 23 | 4 |
| Cryptococcosis | -1 | -1 | -1 | -1 | 1 | -1 | 0 | -1 | -1 | 0 | -6 | 23 | 4 |
| *Vibrio cholerae* | -1 | -1 | -1 | -1 | -1 | 1 | 0 | 0 | -1 | -1 | -6 | 23 | 4 |
| *Chlamydohpila psittaci* | -1 | -1 | -1 | -1 | 0 | 0 | 0 | -1 | 0 | -1 | -6 | 23 | 4 |
| Ockelbovirus (Sindbis) | -1 | -1 | -1 | 1 | -1 | -1 | 0 | -1 | 0 | -1 | -6 | 23 | 4 |
| Parvovirus B19 | 1 | 0 | -1 | -1 | -1 | -1 | 0 | -1 | -1 | -1 | -6 | 23 | 4 |
| BK-virus | 1 | -1 | -1 | -1 | -1 | -1 | 0 | -1 | -1 | -1 | -7 | 15 | 4 |
| JC-virus | 1 | -1 | -1 | -1 | -1 | -1 | 0 | -1 | -1 | -1 | -7 | 15 | 4 |
| Helminths (flat worms) | -1 | -1 | -1 | 0 | -1 | -1 | 0 | -1 | -1 | 0 | -7 | 15 | 4 |
| Helminths (hook worms) | -1 | -1 | -1 | 0 | -1 | -1 | 0 | -1 | -1 | 0 | -7 | 15 | 4 |
| *Entamoeba histolytica* | -1 | -1 | -1 | 0 | -1 | 0 | 0 | -1 | -1 | -1 | -7 | 15 | 4 |
| *Salmonella* Typhi and Paratyphi | -1 | -1 | -1 | -1 | 0 | 1 | -1 | -1 | -1 | -1 | -7 | 15 | 4 |
| Molluscipox virus | 1 | -1 | -1 | -1 | -1 | -1 | 0 | -1 | -1 | -1 | -7 | 15 | 4 |
| *Histoplasma capsulatum* | -1 | -1 | -1 | 0 | -1 | -1 | 0 | -1 | -1 | -1 | -8 | 8 | 4 |
| Hepatitis E virus | -1 | -1 | -1 | -1 | -1 | 0 | 0 | -1 | -1 | -1 | -8 | 8 | 4 |
| *Vibrio* (non-*cholerae*): *V. parahaemolyticus, V. vulnificus and V. cholerae* (non O1 and O139) | -1 | -1 | -1 | -1 | -1 | 0 | 0 | -1 | -1 | -1 | -8 | 8 | 4 |
| *Diphyllobotrium* | -1 | -1 | -1 | -1 | -1 | -1 | 0 | -1 | -1 | -1 | -9 | 0 | 4 |
| Cerkarie dermatitis | -1 | -1 | -1 | -1 | -1 | -1 | 0 | -1 | -1 | -1 | -9 | 0 | 4 |
| Helminths (round worms) | -1 | -1 | -1 | -1 | -1 | -1 | 0 | -1 | -1 | -1 | -9 | 0 | 4 |
| HHV-8 (Kaposi's sarcoma associated) | -1 | -1 | -1 | -1 | -1 | -1 | 0 | -1 | -1 | -1 | -9 | 0 | 4 |
